# Enhancement of *Monascus* yellow pigments production by activating the cAMP signalling pathway in *Monascus purpureus* HJ11

**DOI:** 10.1186/s12934-020-01486-y

**Published:** 2020-12-07

**Authors:** Jiawei Liu, Yun Du, Hongmin Ma, Xiaolin Pei, Mu Li

**Affiliations:** 1 Hubei International Scientific and Technological Cooperation Base of Traditional Fermented Foods, Key Laboratory of Environment Correlative Dietology, College of Food Science and Technology, Huazhong Agricultural University, Hubei Province, Wuhan, 430070 China; 2 Key Laboratory of Combinatorial Biosynthesis and Drug Discovery Ministry of Education, School of Pharmaceutical Sciences, Wuhan University, Wuhan, 430071 China; 3 College of Material, Chemistry and Chemical Engineering, Hangzhou Normal University, Hangzhou, 310012 China

**Keywords:** *Monascus purpureus*, *Monascus* azaphilone pigments, cAMP signaling pathway, cAMP phosphodiesterase, Gene knockout, Fed-batch fermentation

## Abstract

**Background:**

*Monascus* azaphilone pigments (MonAzPs), which were produced by *Monascus* species, have been used as important food colorant and food supplements for more than one billion people during their daily life. Moreover, MonAzPs recently have received more attention because of their diverse physiological activities. However, the high microbial production of MonAzPs is still not always guaranteed. Herein, the aim of this study was to develop an efficient biotechnological process for MonAzPs production.

**Results:**

In this study, exogenous cyclic adenosine monophosphate (cAMP) treatment not only induced MonAzPs production, but also stimulated the expression of a cAMP phosphodiesterase gene, named as *mrPDE*, in *M. purpureus* HJ11. Subsequently, MrPDE was identified as a cAMP phosphodiesterase by in vitro enzymatic reaction with purified enzyme. Further, a gene knockout mutant of *mrPDE* was constructed to verify the activation of cAMP signalling pathway. Deletion of *mrPDE* in *M. purpureus* HJ11 improved cAMP concentration by 378% and enhanced PKA kinase activity 1.5-fold, indicating that activation of cAMP signalling pathway was achieved. The Δ*mrPDE* strain produced MonAzPs at 8563 U/g, with a 2.3-fold increase compared with the WT strain. Moreover, the NAPDH/NADP^+^ ratio of the Δ*mrPDE* strain was obviously higher than that of the wild type strain, which led to a higher proportion of yellow MonAzPs. With fed-batch fermentation of the Δ*mrPDE* strain, the production and yield of MonAzPs achieved 332.1 U/mL and 8739 U/g.

**Conclusions:**

A engineered *M. purpureus* strain for high MonAzPs production was successfully developed by activating the cAMP signalling pathway. This study not only describes a novel strategy for development of MonAzPs-producing strain, but also provides a roadmap for engineering efforts towards the production of secondary metabolism in other filamentous fungi.

## Background

*Monascus* azaphilone pigments (MonAzPs) are large group of secondary metabolites produced via polyketide biosynthesis mainly by *Monascus* spp. strains [1, 2]. MonAzPs have been generally classified into yellow, orange, and red pigment subclasses on the basis of color [3, 4]. MonAzPs have been extensively used as natural food coloring agents and food supplements for more than one billion people [5, 6]. These pigments have also been found to exhibit a wide range of biological activities, such as anti-cancer, anti-inflammation, and anti-obesity [7–9].

The annual MonAzPs production has been estimated to exceed 20,000 metric tons in China alone [6]. Many approaches have been applied for improvement of MonAzPs production, such as mutation breeding, process control, genetic engineering and extraction fermentation [10–14]. Among them, the *Monascus kaoliang* mutation strain produced azaphilone derivatives, such as monascusones A and B, with potential application in sunscreen cosmetics [10]. The highest MonAzPs production was achieved with 211 U/mL using a mutant *M. purpureus* strain M183 in liquid-state (submerged) fermentation [15]. However, these microbial productions of MonAzPs still do not fully meet the demand for cost-effective production.

**Fig. 1:**
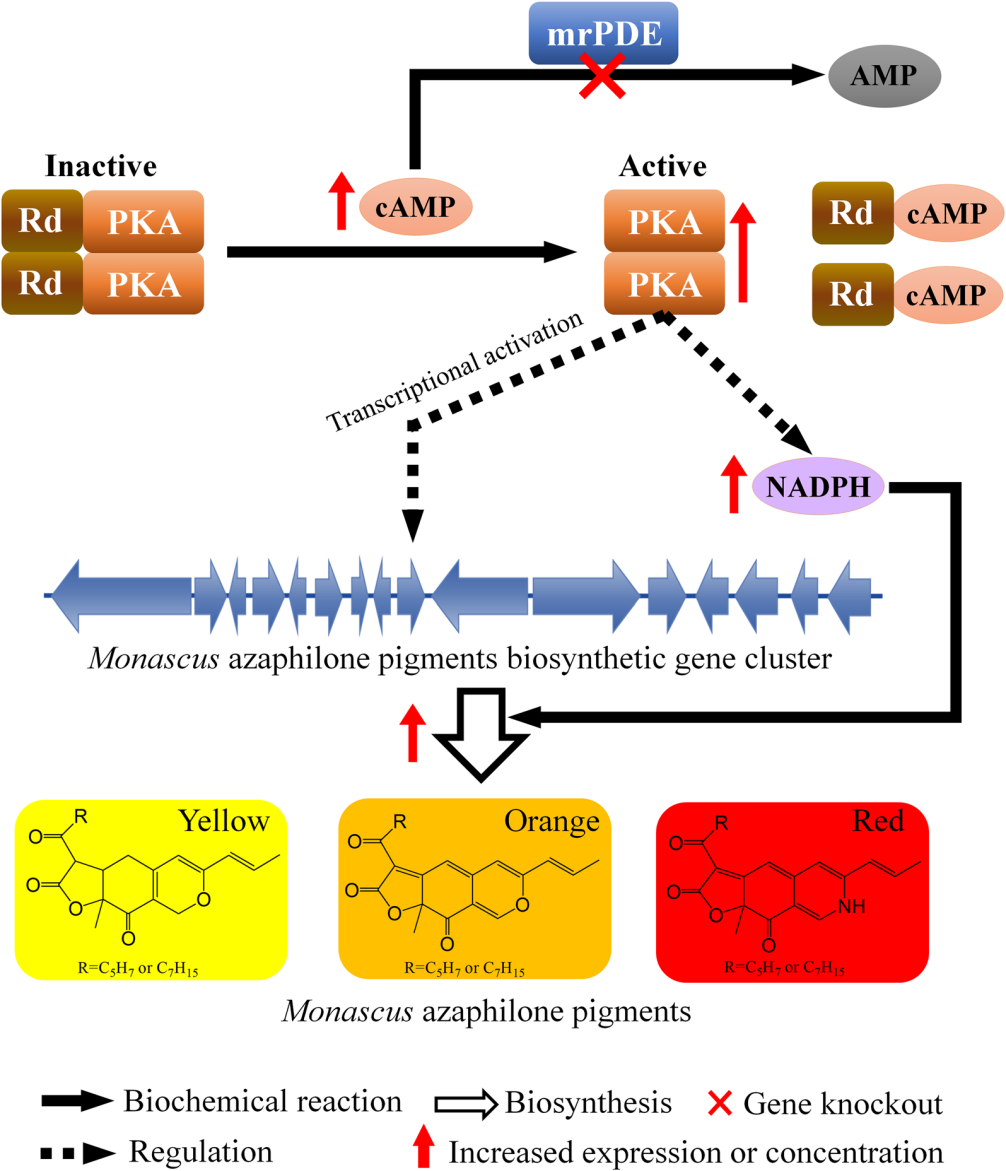
Regulation of cAMP signalling pathway on the MonAzPs biosynthesis in engineered *M. purpureus* HJ11. The concentration of cAMP was negatively regulated by MrPDE. PKA, which was activated by cAMP, modulated the expression of MonAzPs biosynthetic gene cluster indirectly. The enzymes from cluster produced MonAzPs. cAMP, cyclic adenosine monophosphate; PKA, protein kinase A; CA, adenylate cyclase

The cyclic adenosine monophosphate (cAMP) is a second messenger in eukaryotic and prokaryotic cells [16]. cAMP was produced by adenylyl cyclase (AC) with ATP as substrate [17]. The concentrations of cAMP in microbial cells directly governed the activity of protein kinase A (PKA) and indirectly modulated metabolic and transcriptional processes (Fig. 1) [18–20]. The cAMP-PKA pathway is important for various growth and developmental processes in different fungi [21]. Furthermore, cAMP signalling has been implicated in regulating secondary metabolisms in several fungi, including *Fusarium graminearum* and *Aspergillus *species [22, 23]. It has been reported that MonAzPs production in *M. ruber* M7 was improved by addition of exogenous cAMP to the culture medium [24].

In cAMP metabolism, it is rapidly and continuously converted into adenosine 5′ monophosphate (5′-AMP) by cAMP phosphodiesterases (PDEs) [25]. Reduction or elimination of PDE activity could improve cAMP concentration, which led to regulation of secondary metabolites (SMs) biosynthesis [26, 27]. PDE-knockout mutant of *Aspergillus flavus* CA14PTs exhibited twofold higher cAMP level and SM aflatoxin production [28]. In *F. graminearum*, Pde2 was found to be the major PDE responsible for negative regulation of deoxynivalenol production [23]. On the basis of these literatures, it was speculated that knockout of PDE gene in *Monascus* spp. might improve MonAzPs production by increasing cAMP concentration (Fig. 1). However, the PDE gene and its regulation on cAMP in *Monascus* spp. is still unknown.

In this study, a novel PDE gene, *mrPDE*, from *M. purpureus* strain HJ11 was identified by in vitro reaction. Then, the *mrPDE* gene knockout strain was constructed by genetic engineering. The cAMP concentration of ΔmrPDE strain significantly increased, which resulted in a significant improvement of MonAzPs production. Further, a high MonAzPs production was achieved by fed-batch fermentation. This study could provide a new insight for development of efficient MonAzPs-producing strain.

## Methods

### Strains and plasmids

*Monascus purpureus* strain HJ11 was isolated from rice wine starter and preserved in our laboratory. *Escherichia coli* BL21 (DE3) was used as gene expression host. *M. purpureus* HJ11 strain was co-incubated with *Agrobacterium tumefaciens* EHA105, containing recombinant pCAMBIA3300, to obtain deletion mutants. pCAMBIA3300 vector was used as deletion vector and pET30a(+) was used as heterologous gene expression in *E. coli* BL21 (DE3).

### Flask cultivation

*Monascus purpureus* strains were pre-cultured in 10 mL of glucose mineral salt (GM) medium at 30 °C, 120 rpm for 5 days to obtain the seed culture (GM medium in g/L: glucose 30, (NH_4_)_2_SO_4_ 5, KH_2_PO_4_ 5, Na_2_HPO_4_ 3, MgSO_4_ 0.1, CaCl_2_ 0.1, ZnSO_4_·7H_2_O 0.1, FeSO_4_·7H_2_O 0.1, CoSO_4_·7H_2_O 0.05, CuSO_4_·5H_2_O 0.02, and MnSO_4_·H_2_O 0.01). The seed culture was inoculated in 50 mL of GM medium in 250-mL flasks and incubated under same condition for 10 days. When needed, sterile cAMP powder was added to culture medium. The sterile AMP was prepared as following: the cAMP powder (Sigma, Shanghai, China) was dissolved in dd H_2_O to 25 mM, and filtered with sterile 0.22 μm membrane filter for removing microorganism, and then the resulting solution was lyophilized to obtain sterile cAMP powder. The collected mycelia were dried to a constant weight at 80 °C to determine DCW.

The fermentation in rice medium was performed as following: 20 g rice (pre-immersed for 2 h in water at 30 °C), and 50 mL water were put into a 250-mL Erlenmeyer flask, and mixed well. After sterilization at 121 °C for 20 min, 3 mL spore solution (2 × 10^5^/mL) was inoculated, and then incubated at 30 °C for 5–7 days. The spore solution was prepared by washing the mycelium, pre-cultivated on agar for 7 days, with Tween-80 solution (0.5%). The fermented rice was dried to a constant weight at 80 °C and analyzed for pigments.

### Heterologous expression and purification of MrPDE

RNA extraction and cDNA synthesis were performed according to our previous study [29]. The *mrPDE* gene fragment, Monpu1|448456|e_gw1.142.20.1 (ID number of the JGI database), was cloned from cDNA with specificd esigned primers using PCR reaction. The *mrPDE* DNA fragment and vector pET30a(+) were digested by endonucleases EcoRI and HindIII, respectively. The recombinant vector pET30a(+)-*mrPDE* was constructed with T4 DNA ligase and used for expression of mrPDE. All sequences were confirmed by DNA sequencing. The recombinant vector pET30a(+)-*mrPDE* was transformed into *E. coli* BL21(DE3) for expression. The resultant *E. coli* strain was inoculated in 100 mL LB medium, containing 50 μg/mL kanamycin, in 500-mL flask and incubated at 37 °C and 200 rpm. Isopropyl-β-D-thiogalactopyranoside (IPTG) was added to the medium at a final concentration of 0.5 mM, when the optical density of culture reached 0.6–1.0 at 600 nm. Then, the culture was incubated at 18 °C and 200 rpm for 16 h. The His-tagged mrPDE protein was purified using Nickel-NTA Agarose (Qiagen, Valencia, CA, USA), as described in our previous study and was examined on 12% (w/v) SDS-PAGE gel [30]. Protein concentration was estimated by the Bradford method using bovine serum albumin as standard.

### PDE activity detection

Phosphodiesterase activity was determined using a PDE activity assay kit (Colorimetric) following the manufacturer’s instructions (Abcam, Cambridge, United Kingdom) with slight modification [31]. The PDE activities were determined in purified mrPDE and recombinant *E. coli* lysates. The lysates were obtained by disrupting the cells using sonication (W140D, Heat System-Ultrasonics, Inc., NY), followed by centrifugation at 12,000×*g* for 10 min to remove cell debris. One unit of PDE activity for 3′5′-cAMP was defined as the amount of enzyme required to release 1.0 μmol 5′-AMP from 3′5′-cAMP per minute at 30 °C.

### PKA activity determination

Suspended cells were lysed by a high-pressure homogenizer. Cell lysates were incubated at 30 °C as specified by the protocol of PKA (Protein Kinase A) Colorimetric Activity Kit (Thermo Fisher Scientific). The PKA kinase activity was measured by the colorimetric method at 450 nm on a spectrophotometer. The PKA kinase activity was calculated using a calibration curve, which was constructed using five PKA standard solutions.

### qRT-PCR

RNA extraction, cDNA synthesis, and qRT-PCR were performed as previous work described [29]. Briefly, total RNA was extracted from mycelial samples, and then checked for purity and integrity. cDNA synthesis was performed using the PrimeScript RT master mix (TaKaRa) according to the manufacturer instruction. qRT-PCR was conducted with a two-step thermal procedure (step 1, 95 °C for 10 s, and step 2, 40 cycles of 95 °C for 3 s and 60 °C for 25 s) on a 7500 Fast real-time PCR system (Applied Biosystems). The cycle number was used for the quantitation of the expression level. Relative expression level of target cDNA was obtained by the 2^−ΔΔ*CT*^ method via normalization to β-actin (GenBank accession no. AJ417880).

### cAMP concentration measurement

The cAMP concentration was determined according to the previous work with modification [32]. *M. purpureus* HJ11 strains were cultivated in GM medium at 30 °C. Fresh mycelia were harvested, frozen and lyophilized and ground into powder using liquid nitrogen. The powder sample was kept in chilled 6% (w/v) trichloro acetic acid (TCA) solution and incubated for for 20 min. After centrifugation of the mixture at 3500×*g* at 4 °C for 20 min, the supernatant was extracted 3 times with 10 volumes of diethyl ether to remove TCA residues. The resulting extract was dried before further analysis. The cAMP levels were quantified using cAMP Enzyme Immunoassay Kit, Direct (Sigma-Aldrich, St. Louis, MO) following the manufacturer's instructions. In total, assay was repeated three times independently with three biological replicates for each strain.

### NADPH/NADP^+^ ratio determination

*Monascus purpureus* HJ11 strains were cultivated in GM medium at 30 °C. Mycelia were collected after centrifugation and washed in PBS (50 mM, pH 7.0). Mycelia cells were lysed in base solution containing 1% (w/v) dodecyl (trimethyl)azanium bromide. Then, NADP^+^ and NADPH were individually detected according to NADP/NADPH-Glo™ Assay (Promega, Southampton, UK) following the manufacturer’s instructions.

### Batch and fed-batch fermentation

The batch fermentation medium was as follows (g/L): glucose 100, (NH_4_)_2_SO_4_ 30, KH_2_PO_4_ 10, Na_2_HPO_4_ 5, MgSO_4_ 0.5, CaCl_2_ 0.5, ZnSO_4_·7H_2_O 0.3, FeSO_4_·7H_2_O 0.3, CoSO_4_7H_2_O 0.2, CuSO_4_·5H_2_O 0.2, and MnSO_4_·H_2_O 0.1; pH 4.0. The seed culture was prepared by inoculating 10 mL of 5-days pre-culture into 100 mL of GM medium in a 500-mL flask and incubated at 30 °C and 120 rpm for 7 days. The seed culture (300 mL) was transferred to a 10-L stirred tank bioreactor (Baoxing Bioengineering Equipment Co. Ltd, Shanghai, China) containing 6 L of fermentation medium. Fermentation was performed at 30 °C with an agitation speed of 50 to 200 rpm and airflow rate of 1 to 2 vvm. The pH was maintained at 4.0 by automatic addition of 1.0 M NaOH solution.

Similarly, fed-batch fermentation medium was as follows (g/L): glucose 100, (NH_4_)_2_SO_4_ 50, KH_2_PO_4_ 30, Na_2_HPO_4_ 10, MgSO_4_ 1.0, CaCl_2_ 1.0, ZnSO_4_·7H_2_O 1.0, FeSO_4_·7H_2_O 0.5, CoSO_4_·7H_2_O 0.5, CuSO_4_·5H_2_O 0.3, and MnSO_4_·H_2_O 0.1. The seed culture and inoculation were performed similar to the batch fermentation, in a 10-L stirredtank bioreactor (Baoxing Bioengineering Equipment Co. Ltd, Shanghai, China) containing 6 L fermentation media. Fermentation was conducted at 30 °C with an agitation speed of 300 to 800 rpm and an airflow rate of 1.5 to 2.5 vvm. pH was controlled at 4.0 by automatic addition of NaOH solution. After 4 days of fermentation, the residual glucose in medium was determined. When the residual glucose was below 5 g/L, a certain amount (about 300 mL) of feed medium, containing 500 g/L glucose, was added to the 10-L stirred tank bioreactor to maintain glucose concentration at 30 g/L in the fermentation medium, and same amount of culture was withdrawn to analyze residual glucose concentration, biomass and MonAzPs concentration.

### MonAzPs analysis

Intercellular MonAzPs concentration was estimated following our previous method [29]. In this study, the concentration of MonAzPs was measured using UV-1700 spectrophotometer (Shimadzu, Tokyo, Japan) at specific wavelength of 410, 470, and 510 nm that corresponded to the characteristic absorbance of yellow, orange and red pigments, respectively. Total MonAzPs was calculated as the sum of yellow, orange, and red pigments. Statistical analysis was performed using Student's t-test [29].

### Gene knockout in *M. purpureus* HJ11

Targeted gene knockout and complementation of *mrPDE* in *M. purpureus* HJ11 was performed as described in our previous study [29].

### Data availability

Sequences of the genes mentioned in this article are available in GenBank [29].

## Results and discussion

### Improvement in MonAzPs production and *mrPDE* expression by exogenous cAMP addition

**Fig. 2:**
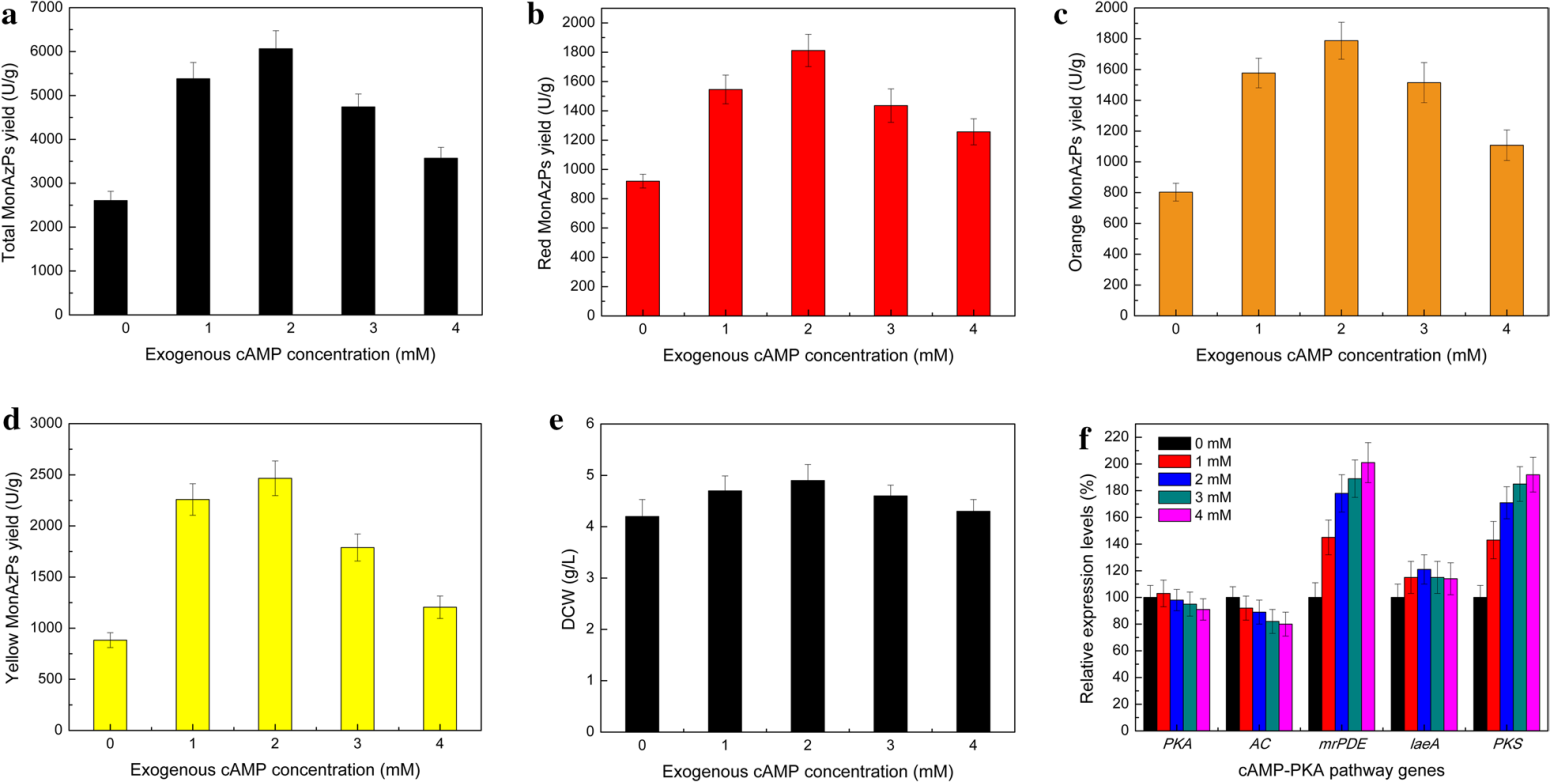
Improvement in MonAzPs production by exogenous cAMP addition. Total MonAzPs (**a**), red MonAzPs (**b**), orange MonAzPs (**b**), and yellow MonAzPs (**d**) yields of *M. purpureus* HJ11 strain at different concentrations of exogenous cAMP in GM medium. **e** DCW of *M. purpureus* HJ11 strain in the presence of exogenous cAMP. Statistical analysis using Student’s *t*-test revealed that there was a significant difference (P < 0.05) between groups (0, 1, 2, 3 and 4 mM). **f** Relative transcription levels of protein kinase A (PKA), adenylate cyclase (AC), mrPDE, LaeA and polyketide synthase (PKS) genes on different exogenous cAMP concentrations. Three replicates were performed for this analysis. Student’s t-test revealed a significant difference in relative expression levels of mrPDE and PKS genes between groups. Groups were not significantly different on PKA, AC and LaeA genes. Error bars represent standard deviations of three flasks

The effect of exogenous cAMP on *M. purpureus* HJ11 was evaluated. The MonAzPs production was determined under different concentrations (0, 1.0, 2.0, 3.0 and 4.0 mM) of cAMP in GM medium. In the presence of exogenous cAMP, the strain showed higher MonAzPs yield (Fig. 2). At 2.0 mM of cAMP concentration, maximum MonAzPs yield of 6065 U/g DCW was achieved, compared to the yield of 2606 U/g DCW without cAMP (Fig. 2a). This was consistent with a previous study, in which addition of 1.0 mM exogenous cAMP promoted MonAzPs yield in *M. ruber* strain M7 [24]. In *Fusarium graminearum*, mycotoxin deoxynivalenol production was approximately increased by 40-folds in cultures treated with 4 mM cAMP [23]. The yields of red, orange, and yellow MonAzPs were 1812 U/g DCW, 1787 U/g DCW and 2466 U/g DCW, respectively (Fig. 2b–d). Among them, maximum rate of increase (179%) was observed in the yield of yellow MonAzPs (Fig. 2d). These results indicated that the cAMP concentration played an important role in the growth and development of fungi and biosynthesis of secondary metabolites. Moreover, it was ascertained that the MonAzPs yield could be improved by increasing the concentration of cAMP.

When the concentration of cAMP was 2.0 mM, maximum dry cell weight (DCW) of 4.6 g/L was achieved, while maximum DCW without cAMP was only 4.2 g/L (Fig. 2e). Similar results were found in *M. ruber* strain M7 [24]. We evaluated the effects of exogenous cAMP on the transcription of genes relative in the cAMP-PKA pathway by quantitative reverse transcription-PCR (qRT-PCR) analysis. There were no marked changes in the expression levels of *mrpigA* and AC genes*.* The expression of global regulator gene *laeA*, which can regulate the MonAzPs biosynthetic gene cluster [14], was a little increased. The MonAzPs polyketide synthase gene *mrpigA*, which was responsible for MonAzPs precursor synthesis [29], showed an obvious increase in expression, explaining the improved production of MonAzPs. Unexpectedly, incubation with cAMP led to a strong induction of expression of a hypothetical PDE gene, named as *mrPDE* (Fig. 2f and Additional file 1: Fig. S1). So, we speculated that this gene was responsible for negative regulation of cAMP in *M. purpureus* HJ11.

### Identification of MrPDE as a cAMP phosphodiesterase

**Fig. 3:**
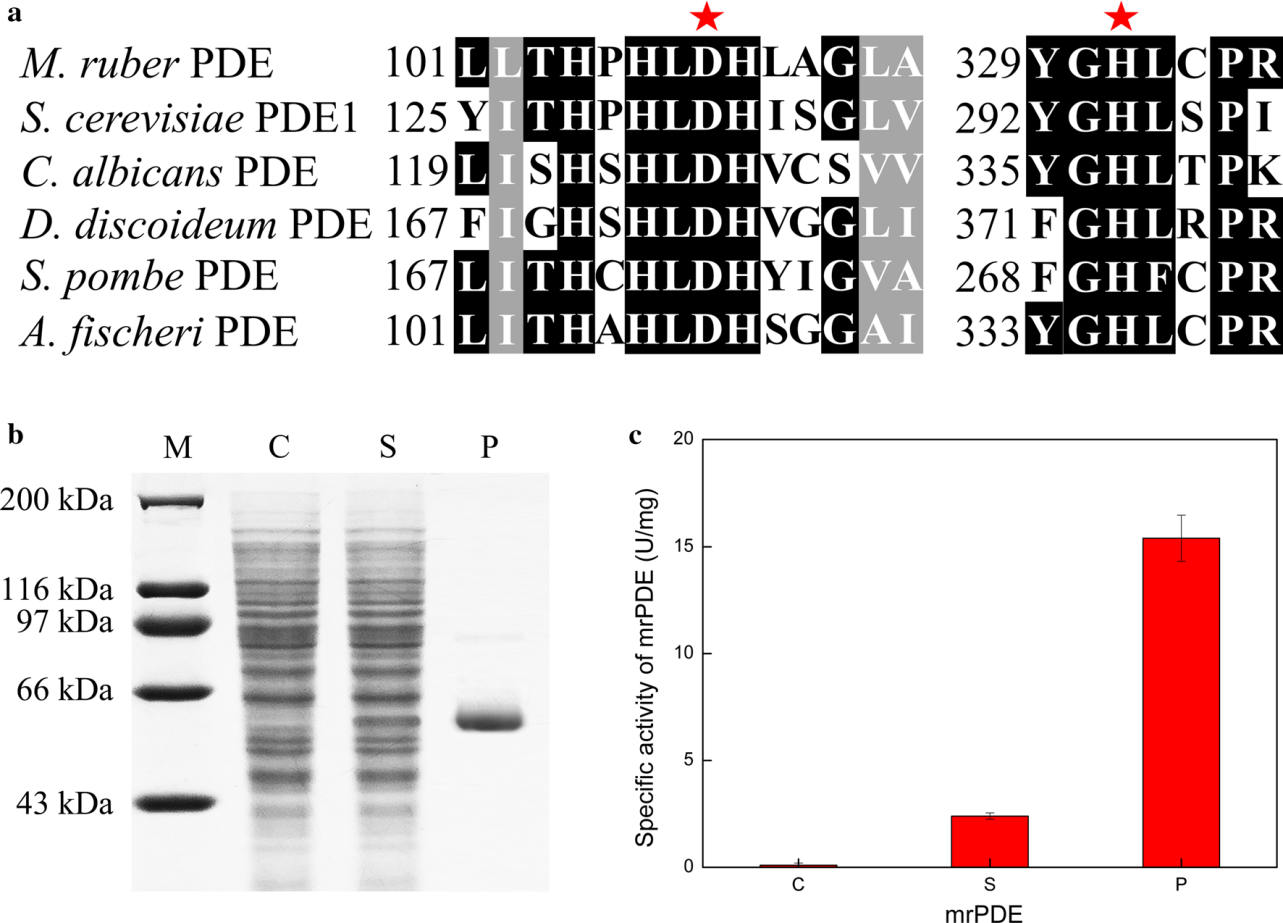
3′,5′-Cyclic nucleotide phosphodiesterase function of MrPDE. **a** Blocks of sequences conserved in MrPDE and several fungal PDEs. Pentagram indicates the possible catalytic amino acid residues. Identical amino acids are on a black background. **b** SDS-PAGE analysis and purification of MrPDE. M lane, protein marker; C lane, supernatant of *E. coli* BL21 (DE3) harboring control pET30a(+); S lane, supernatant of *E. coli* BL21 (DE3) containing pET30a(+)-*mrPDE* induced by IPTG; P lane, purified MrPDE protein. **c** Specific activity of MrPDE. The PDE activity of MrPDE was determined at 30 °C in 50 mM Tris–HCl buffer (pH 8.0) using 3′,5′-cAMP as the substrate. Results were reported as mean value of three replicates ± standard deviation

To the best of our knowledge, there was no report about PDE gene in *Monascus* spp. It was imperative to identify the PDE gene in *M. purpureus* HJ11. Comparison of the amino acid sequence of MrPDE with known sequences in the NCBI protein databases showed that a hypothetical PDE gene, XP_001264269.1 from *Aspergillus fischeri*, displayed the highest similarity of 64% with MrPDE. To further analyze MrPDE, several known PDEs (4OJV_A from *S. cerevisiae*, Q5AGE4 from *Candida albicans*, P12019 from *Dictyostelium discoideum*, and P36599 from *Schizosaccharomyces pombe*) were selected. A multiple alignment of 6 sequences mentioned above was also performed. The alignment analysis revealed two highly conserved amino acid motifs, which contained catalytic residues (Fig. 3a). This result indicated that MrPDE may be a fungal PDE.

To verify the function of MrPDE, the enzymatic activity was evaluated through in vitro reaction. MrPDE was heterologously expressed in *E. coli* BL21(DE3) strain. After induction with 0.5 mM IPTG at 18 °C for 12 h, SDS-PAGE analysis showed that MrPDE was successfully expressed in *E. coli* under the control of the T7 promoter (Fig. 3b, S lane). The enzyme was purified using Ni affinity chromatography column (Fig. 3b, P lane). The catalytic activity of MrPDE was determined with 3′,5′-cAMP as substrate. MrPDE was found to efficiently catalyze the hydrolysis of 3′,5′-cAMP (Fig. 3c). The specific activity of purified MrPDE was 15.4 U/mg, which was close to that of PDE1 (20.5 U/mg) from *S. cerevisiae* S288C [33]. These results indicated that MrPDE was indeed a fungal PDE.

### Activation of cAMP signalling pathway by *mrPDE* knockout

**Fig. 4:**
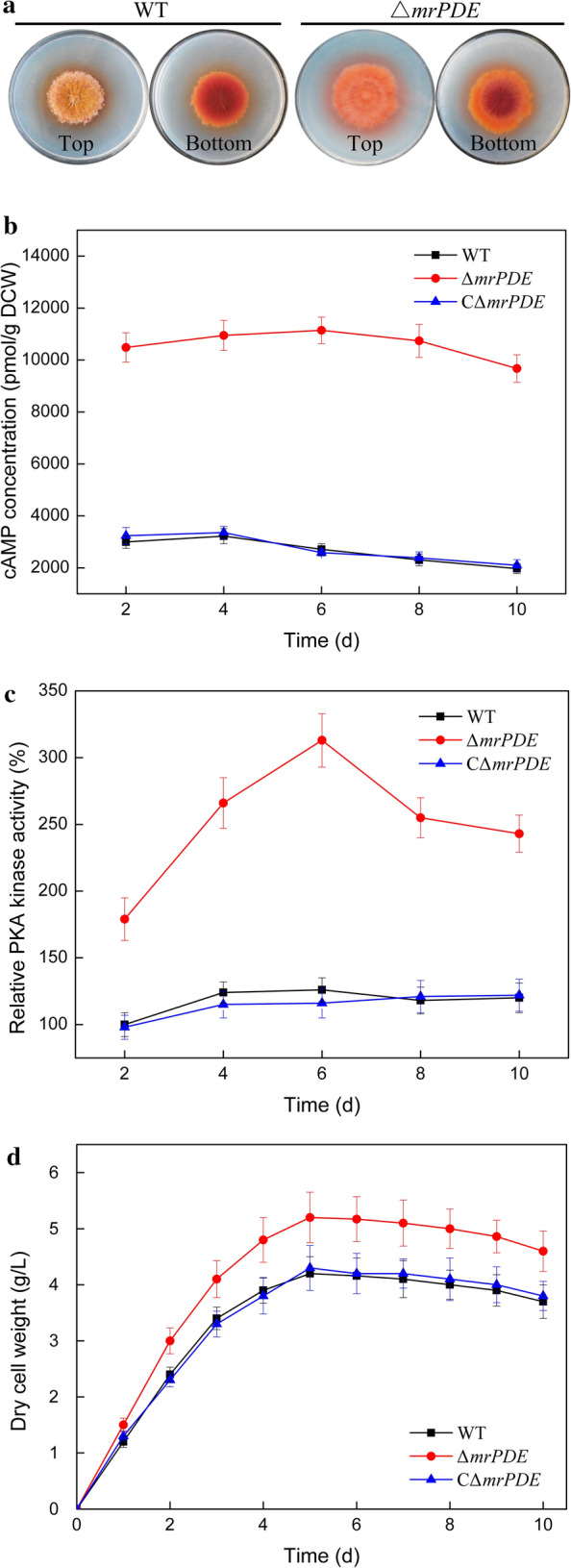
Activation of cAMP signalling pathway by *mrPDE* knockout. **a** Colony morphology of WT and Δ*mrPDE* strains. The strains were cultivated on GM plates at 30 °C for 10 days. The cAMP concentration (**b**), relative PKA kinase activity (**c**), and DCW (**d**) of WT, Δ*mrPDE* and CΔ*mrPDE* strains. Experiments were carried out in three replicates

To construct a MonAzPs high-producing strain, a *mrPDE* gene knockout strain Δ*mrPDE* and a *mrPDE* complemented knockout strain CΔ*mrPDE* were successfully engineered through homologous recombination technology. The colony diameter of Δ*mrPDE* was a little bigger than those of WT and CΔ*mrPDE*, which indicated that the growth of Δ*mrPDE* strain was not inhibited by *mrPDE* gene knockout (Fig. 4a). Meanwhile, the morphology of Δ*mrPDE* colonies grown on GM plate showed more intense color than those of WT and CΔ*mrPDE*, implying that the Δ*mrPDE* strain might have a high production on MonAzPs (Fig. 4a).

The cAMP concentration was determined in each strain at different times. Notably, the cAMP concentration in Δ*mrPDE* strain was significantly higher than those of WT and CΔ*mrPDE* strains, attaining a maximum of 11,145 pmol/g on sixth day (Fig. 4b). The cAMP concentration in WT strain increased to 3219 pmol/g at the fourth day before gradually decreasing (Fig. 4b). Similar cAMP trend was observed in CΔ*mrPDE* strain. This result suggested that knockout of *mrPDE* could inhibit the cAMP degradation, which led to a higher intracellular cAMP concentration. It has been reported that deletion of PDE gene in *Ustilaginoidea virens* and *Magnaporthe oryzae* both resulted in double-fold increase in cAMP concentration [21, 34]. Thus, it was ascertained that deletion of PDE gene could efficiently increase cAMP concentration in *M. purpureus* HJ11.

In cAMP signalling pathway, PKA activity is essential for regulation of primary and secondary metabolism [35, 36]. Our data showed that knockout of *mrPDE* could induce PKA kinase activity (Fig. 4c), suggesting the increase of cAMP concentration triggered PKA activation [37]. The DCW of Δ*mrPDE* strain reached 5.1 g/L, which was higher than those of WT (4.2 g/L) and CΔ*mrPDE* strains (4.2 g/L) (Fig. 4d). These results indicated that the cAMP signalling pathway was activated by knockout of *mrPDE*.

### Improvement of MonAzPs yield in Δ*mrPDE* strain

**Fig. 5:**
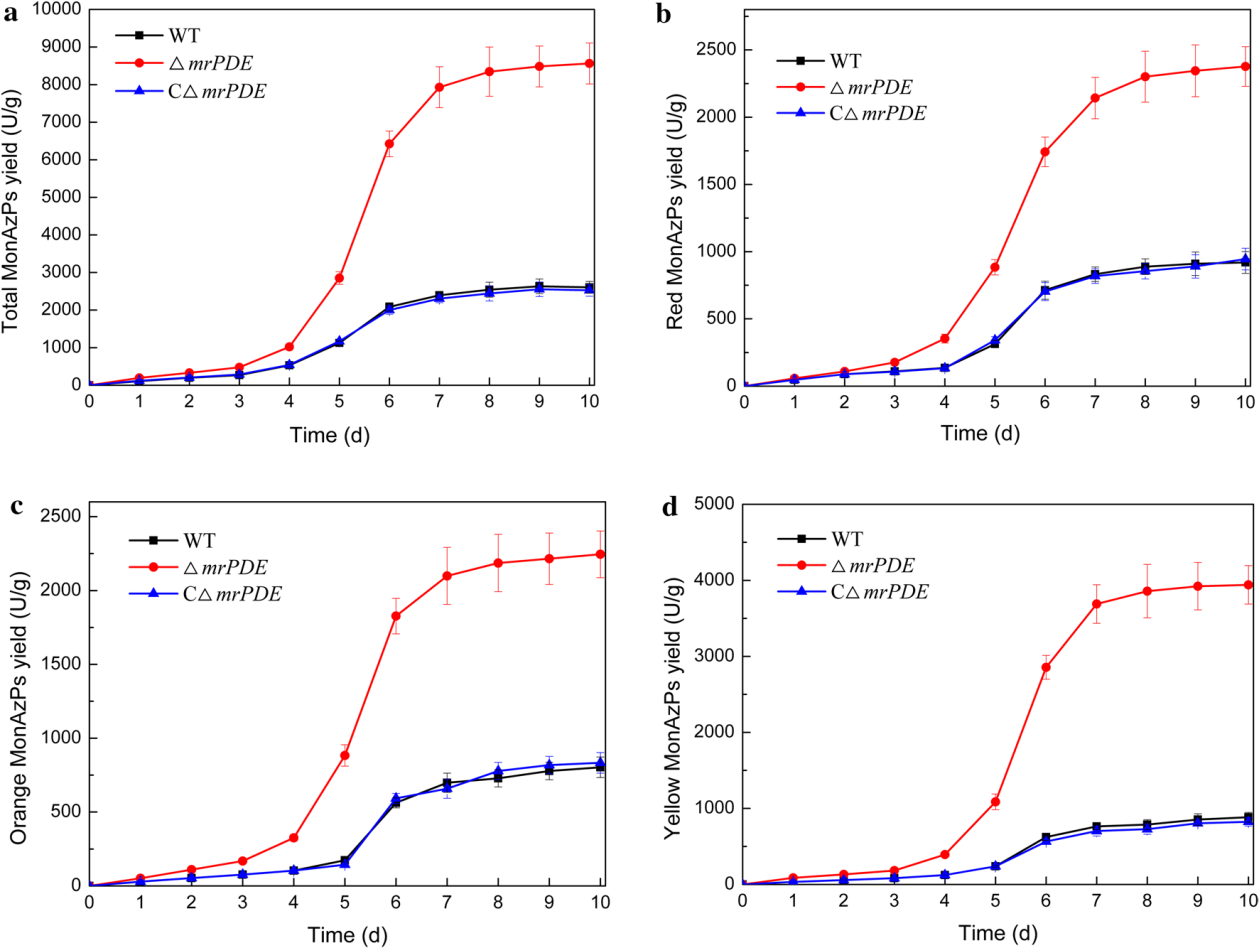
Comparison of MonAzPs production between the WT, Δ*mrPDE*, and CΔ*mrPDE* strains. Time courses of total MonAzPs (**a**), red MonAzPs (**b**), orange MonAzPs (**c**), yellow MonAzPs (**d**) production of WT, Δ*mrPDE* and CΔ*mrPDE* strains, respectively. The strains were cultivated in GM medium at 30 °C for 10 days. The means and standard deviations calculated from measurements from three biological replicates for each strain were shown

For further confirmation of the MonAzPs production in Δ*mrPDE* strain, shake-flask fermentation was performed. After fermentation for 10 days, the MonAzPs yield of Δ*mrPDE* strain reached 8563 U/g DCW, which was 2.3-fold higher than that of WT strain (Fig. 5). The yields of red, orange, and yellow pigments of Δ*mrPDE* strain were 2377, 2245 and 3941 U/g DCW, respectively (Fig. 5b–d). These yields were 1.58-times, 1.80-times, and 3.46-times higher than those of WT strain, respectively. The *Monascus purpureus* WT and Δ*mrPDE* strains were also cultivated in rice medium. The yields of red, orange, and yellow pigments in Δ*mrPDE* strain were 3721, 2655 and 4864 U/g DCW, respectively, which were higher than those of WT strain (2924, 2387 and 4059 U/g DCW). Similar study has been reported in *F. graminearum*, the knockout of PDE gene *pde1* resulted in increased production of secondary metabolite deoxynivalenol [23]. The deletion of PDE gene *pdeH* from *A. flavus* led to an increased production of aflatoxin to 110 mg/mL from 48 mg/mL [38].

To explain the reason of increasing MonAzPs yield, the expression of each genes from *Monascus* azaphilone pigments biosynthetic gene cluster (MPBGC) was determined by quantitative reverse transcription-PCR (qRT-PCR) analysis. All MPBGC genes were expressed in the WT and Δ*mrPDE* strains. Unexpectedly, as shown by the Δ*mrPDE* strain, transcriptional activation of large parts of the genes in the MABGC was achieved, apart from *mrpigH*, *mrpigI*, *mrpigL* and *mrpigP*, which didn’t participate in MonAzP biosynthesis (Additional file 1: Fig. S2) [29, 39]. The downstream targets of PKA include transcriptional regulators and other effectors to control gene transcriptional expression [40]. These suggested that PKA, activated by cAMP, improved the transcriptional expression of MPBGC genes through indirect activation [41].

It is worth noting that the increased proportion of yellow MonAzPs yield was significantly higher than that of total MonAzPs yield in Δ*mrPDE* strain (Figs. 2 and 5). In our previous study, orange MonAzPs were found to be converted into yellow MonAzPs in the presence of adequate NADPH [42]. So, it was speculated that the increased cAMP concentration led to higher rate of NADPH/NADP^+^. There is little knowledge about the influence of cAMP on NADPH/NADP^+^ rate in microorganism. Herein, it was found that the ratio in Δ*mrPDE* strain was 0.91, which are much higher than those of WT and CΔ*mrPDE* strains (0.55 and 0.54, respectively) (Additional file 1: Fig. S3). This might be the main reason for the significantly higher yield of yellow MonAzPs than those of red and orange MonAzPs.

### High-density fermentation for high MonAzPs production

**Fig. 6:**
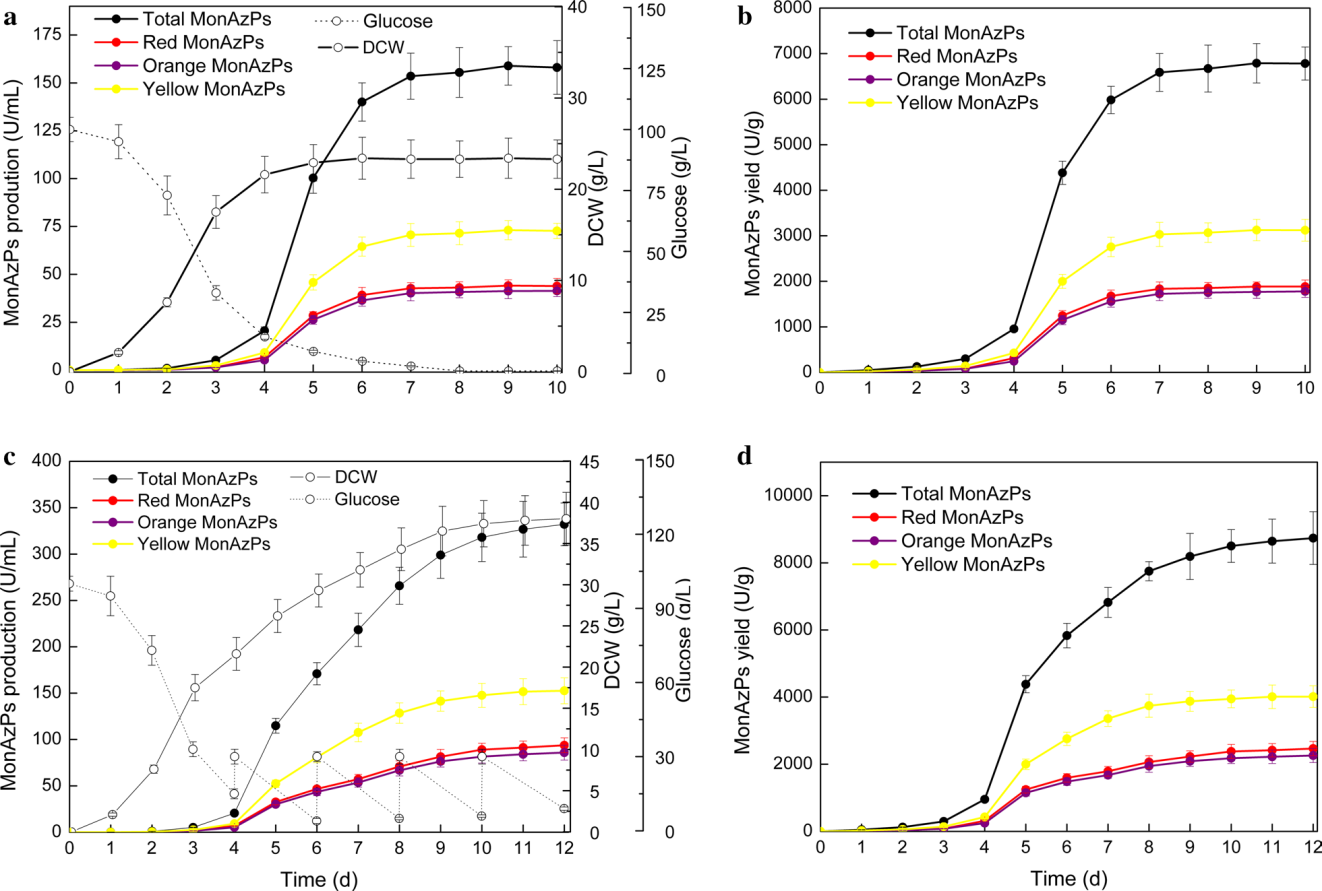
MonAzPs production in batch and fed-batch fermentation using Δ*mrPDE* strain. Time course of cell growth, residual glucose and MonAzPs production during batch fermentation (**a**) and fed-batch fermentation (**c**). Patterns of MonAzPs yield in batch fermentation (**b**) and fed-batch fermentation (**d**). Data are presented as mean ± SD from three independent experiments of three replicates each (n = 9)

In order to enhance the production of MonAzPs, batch fermentation of Δ*mrPDE* strain was performed in a medium, containing initial glucose concentration of 100 g/L. During the fermentation process, *M. purpureus* WT and Δ*mrPDE* strains formed pellet with an average diameter of 3.7 ± 0.2 mm. The formation of pellet contributed to pigments production. After 10 days of cultivation, total MonAzPs production reached a maximum of 158.9 U/mL (Fig. 6a), and corresponding total MonAzPs yield was 6782 U/g (Fig. 6b). However, this yield was lower than that in shake flask (8563 U/g). This might be attributed to the insufficient glucose supply (< 5 g/L) in the middle and later stages of fermentation, where mycelia accumulated the main part of MonAzPs (Fig. 6a).

For improvement of glucose supply, a fed-batch fermentation was also performed in a 10-L stirred fermenter [43]. The glucose solution was added in fermentation medium to maintain the glucose at 30 g/L when residual glucose was below 5 g/L. The Δ*mrPDE* strain displayed rapid growth, and DCW reached 38.1 g/L, which was significantly higher than that of batch fermentation (Fig. 6c). In another study, fed-batch fermentation of *Monascus anka* strain GIM 3.592 achieved a DCW of 39.77 g/L [44]. Subsequently, a significant increase in MonAzPs production was observed, reaching a maximum of 332.1 U/mL. The total MonAzPs yield reached 8739 U/g DCW, slightly higher than that in shake flask fermentation (Fig. 6d). It has been reported that a mutant *M. purpureus* strain M183 produced MonAzPs at 211.6 U/mL [15]. To the best of our knowledge, this study achieved highest MonAzPs yield and production.

## Conclusion

In summary, *mrPDE* was identified as a PDE from *M. purpureus* HJ11. Subsequently, knockout of the *mrPDE* gene was performed to enhance cAMP concentration. The MonAzPs yield in Δ*mrPDE* strain was achieved at 8563 U/g, which was 2.3-folds higher than that of WT strain. In order to improve the MonAzPs production, the fed-batch fermentation was performed in a 10-L stirred fermenter. The MonAzPs production was significantly enhanced to 332.1 U/mL, with the MonAzPs yield of 8739 U/g DCW. This study describes a promising method for high production of MonAzPs and provides a strategy for metabolic engineering of secondary metabolism in other filamentous fungi.

## Supplementary Information

**Additional file 1: Fig. S1.** Relative expression levels of *mrPDE* gene with or without 2.0 mM cAMP during the cultivation. Error bars represent standard deviations of three flasks. Three replicates were performed for this analysis. **Fig. S2.** qRT-PCR analysis of the MPBGC genes of the *M. purpureus* HJ11 wild-type (WT) and Δ*mrPDE* knockout strains. Gene expression levels from WT strain are taken as the basis of comparison, with the means and standard deviations calculated from measurements from three biological replicates. **Fig. S3.** NADPH/NADP+ ratio analysis of *M. purpureus* HJ11 WT and Δ*mrPDE* knockout strains. NADP+ and NADPH were individually detected. The NADPH/NADP+ ratio was calculated with NADP+ and NADPH levels.

## Data Availability

The datasets used and analyzed during the current study are available from the corresponding author on reasonable request.
