# Supplementary material for: Enhancement of Monascus yellow pigments production by activating the cAMP signalling pathway in Monascus purpureus HJ11
Source: Microb Cell Fact. 2020 Dec 7;19:224. doi: 10.1186/s12934-020-01486-y (PMC7720387; doi:10.1186/s12934-020-01486-y)
Supplement: Supplementary file 1 — Additional file 1: Fig. S1. Relative expression levels of mrPDE gene with or without 2.0 mM cAMP during the cultivation. Error bars represent standard deviations of three flasks. Three replicates were performed for this analysis. Fig. S2. qRT-PCR analysis of the MPBGC genes of the M. purpureus HJ11 wild-type (WT) and ΔmrPDE knockout strains. Gene expression levels from WT strain are taken as the basis of comparison, with the means and standard deviations calculated from measurements from three biological replicates. Fig. S3. NADPH/NADP+ ratio analysis of M. purpureus HJ11 WT and ΔmrPDE knockout strains. NADP+ and NADPH were individually detected. The NADPH/NADP+ ratio was calculated with NADP+ and NADPH levels. [file 12934_2020_1486_MOESM1_ESM.docx]

**Additional file**

Enhancement of *Monascus* azaphilone pigments production by activating the cAMP signalling pathway in *Monascus purpureus* HJ11

Jiawei Liu^1^, Yun Du^1^, Hongmin Ma^2^, Xiaolin Pei^3^, Mu Li^1,^*

^1^ Hubei International Scientific and Technological Cooperation Base of Traditional Fermented Foods, Key Laboratory of Environment Correlative Dietology, College of Food Science and Technology, Huazhong Agricultural University, Wuhan, Hubei Province, 430070, China

^2^ Key Laboratory of Combinatorial Biosynthesis and Drug Discovery Ministry of Education, School of Pharmaceutical Sciences, Wuhan University, Wuhan 430071, China

^3^ College of Material, Chemistry and Chemical Engineering, Hangzhou Normal University, Hangzhou, 310012, PR China

^*^ Corresponding authors: Mu Li, limu@mail.hzau.edu.cn; Fax: +86-27-87282111

3 Figures





**Fig. S1** Relative expression levels of *mrPDE* gene with or without 2.0 mM cAMP during the cultivation. Error bars represent standard deviations of three flasks. Three replicates were performed for this analysis.





**Fig. S2** qRT-PCR analysis of the MPBGC genes of the *M. purpureus* HJ11 wild-type (WT) and Δ*mrPDE* knockout strains. Gene expression levels from WT strain are taken as the basis of comparison, with the means and standard deviations calculated from measurements from three biological replicates.





**Fig. S3** NADPH/NADP^+^ ratio analysis of *M. purpureus* HJ11 WT and Δ*mrPDE* knockout strains. NADP^+^ and NADPH were individually detected. The NADPH/NADP^+^ ratio was calculated with NADP^+^ and NADPH levels.
